# Bone marrow transplantation stimulates neural repair in Friedreich's ataxia mice

**DOI:** 10.1002/ana.25207

**Published:** 2018-04-23

**Authors:** Kevin C. Kemp, Kelly Hares, Juliana Redondo, Amelia J. Cook, Harry R. Haynes, Bronwen R. Burton, Mark A. Pook, Claire M. Rice, Neil J. Scolding, Alastair Wilkins

**Affiliations:** ^1^ Multiple Sclerosis and Stem Cell Group, Translational Health Sciences, Bristol Medical School University of Bristol Bristol United Kingdom; ^2^ Department of Cellular Pathology North Bristol National Health Service Trust Bristol United Kingdom; ^3^ Infection and Immunity, School of Cellular and Molecular Medicine University of Bristol Bristol United Kingdom; ^4^ Synthetic Biology Theme, Institute of Environment, Health and Societies, Biosciences, Department of Life Sciences, College of Health and Life Sciences Brunel University London London United Kingdom

## Abstract

**Objective:**

Friedreich's ataxia is an incurable inherited neurological disease caused by frataxin deficiency. Here, we report the neuroreparative effects of myeloablative allogeneic bone marrow transplantation in a humanized murine model of the disease.

**Methods:**

Mice received a transplant of fluorescently tagged sex‐mismatched bone marrow cells expressing wild‐type frataxin and were assessed at monthly intervals using a range of behavioral motor performance tests. At 6 months post‐transplant, mice were euthanized for protein and histological analysis. In an attempt to augment numbers of bone marrow–derived cells integrating within the nervous system and improve therapeutic efficacy, a subgroup of transplanted mice also received monthly subcutaneous infusions of the cytokines granulocyte‐colony stimulating factor and stem cell factor.

**Results:**

Transplantation caused improvements in several indicators of motor coordination and locomotor activity. Elevations in frataxin levels and antioxidant defenses were detected. Abrogation of disease pathology throughout the nervous system was apparent, together with extensive integration of bone marrow–derived cells in areas of nervous tissue injury that contributed genetic material to mature neurons, satellite‐like cells, and myelinating Schwann cells by processes including cell fusion. Elevations in circulating bone marrow–derived cell numbers were detected after cytokine administration and were associated with increased frequencies of Purkinje cell fusion and bone marrow–derived dorsal root ganglion satellite‐like cells. Further improvements in motor coordination and activity were evident.

**Interpretation:**

Our data provide proof of concept of gene replacement therapy, via allogeneic bone marrow transplantation, that reverses neurological features of Friedreich's ataxia with the potential for rapid clinical translation. Ann Neurol 2018;83:779–793

Friedreich's ataxia (FA) is an autosomal recessive inherited ataxia caused, in >95% of cases, by a homozygous GAA.TTC trinucleotide repeat expansion within intron 1 of the *FXN* gene.^1^ This triplet expansion results in transcriptional repression of frataxin,^2^ a small mitochondrial protein involved in iron–sulfur cluster biosynthesis. Typically, patients with the condition experience insidious accumulation of neurological disability characterized pathologically by lesions in the dorsal root ganglia (DRG), sensory peripheral nerves, spinal cord, and cerebellar dentate nucleus.^3^, ^4^

Neuronal atrophy and dysfunctional glia are both thought to contribute to neuropathology in FA.^3^, ^5^, ^6^, ^7^ Despite advances in understanding of the disease, current therapeutics show little ability to protect nervous tissue and no capacity to promote repair. Adult stem cell populations, notably those that reside within the bone marrow (BM), have been shown both to provide neurotrophic support and to contribute to neuronal/glial cell types in the brain through processes likely involving cellular fusion.^8^, ^9^, ^10^, ^11^, ^12^, ^13^ The observation that BM cells can migrate and integrate within the nervous system, and persist apparently for decades,^8^, ^9^ may offer a biological mechanism that can be exploited therapeutically.^12^, ^13^ Utilizing allogenic BM transplantation (BMT) as a mode of gene therapy, to provide a source of "genetically normal" donor cells to access affected tissue and support endogenous cells of the central and peripheral nervous system, may afford significant therapeutic potential,^14^, ^15^ particularly in a multi‐system disease such as FA.

We have recently described the neuroprotective properties of both granulocyte‐colony stimulating factor (G‐CSF) and stem cell factor (SCF) in a murine model of FA,^16^ two agents used in clinical practice to mobilize BM stem cells prior to a peripheral blood (PB) stem cell harvest.^17^, ^18^ In both healthy animals and animals with central nervous system (CNS) injury, the numbers of BM‐derived cells detectable in the brain are increased following treatment with G‐CSF and SCF.^19^, ^20^ This implies that migration of BM‐derived cells into the nervous system has potential for therapeutic manipulation, and in addition to their neuroprotective effects in FA,^16^ G‐CSF and SCF may also aid the delivery of BM cells to sites of injury in the disease, stimulating neural repair.

Here, we explore whether myeloablative allogeneic BMT of cells expressing the wild‐type *Fxn* gene can be harnessed as a potential neuroreparative gene therapy for FA; and secondly, to extend our previous studies, whether subsequent administration of G‐CSF and SCF can enhance BM‐derived cell integration within the diseased nervous system and improve therapeutic efficacy.

## Materials and Methods

### Experimental Design

**Figure 1:**
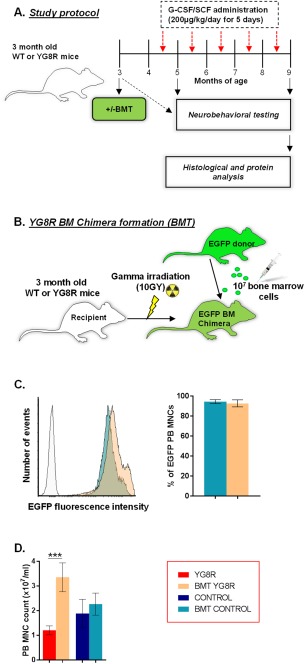
Myeloablative allogeneic bone marrow (BM) transplantation (BMT) and BM chimerism in YG8R mice. (A) Experimental protocol using wild‐type (WT) and YG8R mice to investigate the effects of allogeneic BMT. At 3 months of age, mice were assessed using an extensive range of behavioral performance tests and subsequently given a BMT from a ubiquitously expressing enhanced green fluorescent protein (EGFP) donor. After 8 weeks, mice were again assessed at monthly time points using behavioral performance tests. A subset of transplanted YG8R mice were also given monthly infusions of granulocyte‐colony stimulating factor (G‐CSF) and stem cell factor (SCF; *red arrows*). At 9 months of age, mice were euthanized for histological analysis. (B) Three‐month‐old recipient mice were given an allogeneic BMT to create EGFP‐expressing BM chimeras. Mice were lethally irradiated, with a single dose of 1,000rad, 6 hours prior to receiving 1 × 10^7^ unfractionated BM EGFP‐expressing cells by tail vein injection. (C) Flow cytometric analysis (histogram) to determine the level of BM chimerism within EGFP BM cell–transplanted mice 6 weeks post‐transplant. The percentage of peripheral blood (PB) mononuclear cells (MNCs), harvested from transplanted mice, that were positive for EGFP was calculated (MNCs with a relative fluorescence higher than that of cells derived from non‐transplanted WT control mice [white peak]). (D) The PB MNC counts of both non‐transplanted and transplanted WT control and YG8R mice. Statistical comparisons of control versus YG8R, YG8R versus BMT YG8R, and control versus BMT control mice were analyzed using analysis of variance followed by Holm–Sidak multiple comparisons test. ***p < 0.001. Values represent mean ± standard error of the mean. For all tests, n = 4 (BMT YG8R), all other groups n = 5.

Both wild‐type control mice and YG8R mice received a myeloablative allogeneic BMT to produce transplanted wild‐type controls (BMT control) and transplanted YG8R mice (BMT YG8R). A subgroup of BMT YG8R mice also received monthly infusions of G‐CSF/SCF (BMT YG8R G‐CSF/SCF). Experimental protocols are described in Figure 1A and B. Sample sizes were based on our previous reports using the YG8R model.^16^

### Animals

All animal experiments were performed in accordance with the UK Animals (Scientific Procedures) Act 1986 and approved by the University of Bristol Animal Welfare and Ethical Review Body. Female *Fxn* ^*tm1Mkn*^ Tg(FXN)YG8Pook/J (YG8R) transgenic mice, which carry a human genomic *FXN* transgene (on a murine frataxin null background) containing expanded GAA repeats of 82 to 190 units within intron 1 of *FXN*, were used. (Sex differences in disease trajectory are reported in YG8R mice; female YG8R mice show a stronger behavioural phenotype and reduced frataxin protein in the brain, when compared to male YG8R mice, that more closely resembles human disease.^21^) Both ataxic (strain # *Fxn* ^*tm1Mkn*^ Tg[FXN]YG8Pook/J, stock # 008398) and transgenic mice ubiquitously expressing enhanced green fluorescent protein (EGFP; strain # C57BL/6‐Tg[CAG‐EGFP]131Osb/LeySopJ, stock # 006567) were purchased from The Jackson Laboratory (Bar Harbor, ME). Control C57BL/6 VAF/Elite mice were provided by Charles River UK (Margate, UK). Genotyping was performed by The Jackson Laboratory to confirm the genetic background of all YG8R mice, and upon arrival, mice were randomly assigned to a treatment group. Mice were housed in a pathogen‐free facility, with free access to sterile food and water.

### BMT: Generation of EGFP‐Expressing BM Chimeric Mice

Donor BM cells were harvested, under sterile conditions, from 10‐ to 12‐week‐old male C57BL/6 EGFP‐expressing transgenic mice.^22^ Twelve‐week‐old female recipient mice (control and YG8R mice) were lethally irradiated, with a single dose of 1,000rad from a Cesium‐137 source, 6 hours prior to receiving 1 × 10^7^ unfractionated EGFP‐expressing donor BM cells by tail vein injection. Founder mice of both parent lines used to generate the YG8R mouse had been back‐crossed to C57BL/6 for 5 generations^23^, ^24^; thus, no immunosuppression was provided post‐transplant.

Wild‐type BM donor mice were male, and recipients (control and YG8R mice) were female. This allowed nuclear material from the male donor cells to be traced within the recipient using the male Y chromosome.

Myeloablative BMT can be associated with significant morbidity.^25^ All transplanted mice were therefore left to recover for 6 weeks prior to any further experimentation. For the total duration of the experiment, 2 mice within the BMT control group were removed from the study due to transplant‐related complications. No complications were observed in the YG8R mice. A single YG8R mouse assigned to the BMT YG8R group was removed from the study due to illness (unrelated to its genetic phenotype), before any experimental intervention, on veterinary advice. BMT was shown to have statistically significant (*p* < 0.05) negative effects on the weight of both YG8R and control mice (data not shown).

### PB Mononuclear Cell Counts and Detection of Chimerism

At 6 and 20 weeks post‐BMT, hematopoietic reconstitution was evaluated in PB by flow cytometry (FACSCalibur; Becton Dickinson, Franklin Lakes, NJ). Briefly, 100 µl PB was harvested from the tail vein and suspended in phosphate‐buffered saline (PBS) pH 7.4/ethylenediaminetetraacetic acid (2mg/ml). Red cells were removed using red cell lysis buffer, and the remaining nucleated cell population was resuspended in PBS/3% fetal bovine serum, counted using a hemocytometer, and examined for EGFP expression when excited at 488nm using flow cytometric analysis. PB harvested from a non‐transplanted C57BL/6 mouse was used as a reference control. Data were evaluated using Becton Dickinson CellQuest software.

### Cytokine Administration

Cytokines doses were based on standard regimens for G‐CSF mobilization of PB hematopoietic stem cells in mice.^16^, ^26^ Mice received subcutaneous injection of murine G‐CSF and SCF (PeproTech, Rocky Hill, NJ; both 200 µg/kg) in PBS once daily for 5 consecutive days. Treatments were repeated monthly. PBS alone was administered as a vehicle control.

### Neurobehavioral Testing

Body weight, rotarod, grip strength, string test, beam‐walk test, open field, and gait analysis were recorded monthly between 3 and 9 months of age as previously described.^16^

### Tissue Preparation (Histology)

Mice were anaesthetized by intraperitoneal injection of Euthatal and perfused with PBS followed by 4% paraformaldehyde in PBS. The brains, spinal cords, and DRG were dissected, placed in 4% paraformaldehyde in PBS for 24 hours at 4°C, processed, and subsequently embedded in paraffin for sectioning on a rotary microtome (LM2135; Leica Microsystems, Wetzlar, Germany) and mounting on glass slides.

### Immunohistochemistry and Imaging

Immunofluorescent labeling and microscopic imaging has been described previously.^16^ Primary antibodies used were ßIII‐tubulin (ab78078, 1:250; Abcam, Cambridge, MA), calbindin‐D28K (C2724, 1:500; Sigma‐Aldrich, St Louis, MO), CD11b/c (ab1211, 1:100, Abcam), EGFP (ab6556, 1:500, Abcam), glial fibrillary acidic protein (GFAP; ab33922, 1:200, Abcam), IBA‐1 (ab5076, 1:200, Abcam), NeuN (ab177487, 1:500 and ab104224, 1:500, Abcam), S100 (MAB079, 1:200; Millipore, Billerica, MA), S100 (Z0311, 1:200; Dako, Carpinteria, CA). Secondary antibodies were Alexa Fluor 488/555, goat/donkey anti‐mouse (1:500), Alexa Fluor 488/555, goat/donkey anti‐rabbit (1:500), and Alexa Fluor 488/555, donkey anti‐goat (1:500; Invitrogen, Carlsbad, CA). Sections were mounted in VECTASHIELD medium containing the nuclear dye 4′,6′‐diamidino‐2‐phenylindole (Vector Laboratories, Burlingame, CA).

### Fluorescent In Situ Hybridization

Sections (8 µm) were processed and labeled as previously described.^27^ Fluorescent in situ hybridization (FISH) probes (mouse: chromosomes X [locus: Xqc3, green 5‐fluorescein 2′‐deoxyuridine 5′‐triphosphate (dUTP)] and Y control probe [locus: Y, red 5‐ROX dUTP]; Empire Genomics, Buffalo, NY) were applied directly to the tissue sections. DNA was denatured at 83°C for 5 minutes and then renatured with FISH probes by incubating overnight at 37°C.

Using epifluorescence microscopy, each section was scanned for Purkinje cell or DRG neurons containing the Y chromosome. Each cell was subsequently scanned using confocal microscopy acquiring 0.1‐ to 0.2 µm serial sections throughout the entire cell soma. All Z‐stack and 3‐dimensional imaging was created using both Leica Application Suite Advanced Fluorescence software and Volocity 3D image software (PerkinElmer, Waltham, MA).

### Histological Staining

For histological assessment, tissues were sectioned, deparaffinated, rehydrated, and stained with hematoxylin and eosin (visualization of DRG vacuoles).

### Cell Quantification/Measurements

Cell numbers, DRG vacuole quantification, and neuronal cell size measurements were recorded as previously described.^16^

### Binucleate Cells

Fusion of BM‐derived cells with other cell types reveals the formation of binucleate heterokaryons; thus, identification of binucleate cells in the nervous system can be used as an indirect measure of these cell fusion events.^28^ Each section was scanned along either the entire length of the Purkinje cell layer or the cross‐sectional area of the DRG/cerebellar dentate nucleus, for neuronal cell bodies containing 2 separate nuclei. A minimum of 1,000 DRG neurons, 1,000 Purkinje neurons, or 500 cerebellar dentate nucleus neurons from each mouse were examined.

### Protein Analysis

Proteins were isolated from microdissected formalin‐fixed, paraffin‐embedded (FFPE) cerebellum sections using the Qproteome FFPE Tissue kit (Qiagen, Valencia, CA) according to the manufacturers' instructions. Subsequent Western blotting of protein lysates and densitometric analysis were carried out as previously described.^29^ Primary antibodies used were anti‐β actin (ab8227, 1:5,000, Abcam), anti‐catalase (C0979, 1:2,000, Sigma‐Aldrich), anti‐human frataxin (ab110328, 1:2,000, Abcam), anti‐frataxin (ab113691, 1:2,000, Abcam), anti–glutathione peroxidase‐1 (ab22604, 1:1,000, Abcam), anti‐NeuN (ab177487, 1:5,000, Abcam), anti–nuclear factor (erythroid‐derived 2)‐like 2 (Nrf2; sc‐722, 1:3,000; Santa Cruz Biotechnology, Santa Cruz, CA), anti‐peroxisome proliferator‐activated receptor gamma coactivator 1‐alpha (PGC‐1alpha) (sc‐13067, 1:3,000, Santa Cruz Biotechnology), anti‐SOD1 (ab16831, 1:5,000, Abcam), and anti‐SOD2 (ab16956, 1:5,000, Abcam). (Of note, increases in total frataxin are likely to be underestimated when compared to human frataxin due to differences in antibody (ab113691, Abcam) species reactivity; the human protein reactivity of the antibody is approximately 50% of mouse protein reactivity at the same concentration. Commercial mouse‐specific frataxin antibodies are not available.)

### Statistical Analysis

Those analyses that did not employ longitudinal replicates from the same animal were performed using Prism (GraphPad, La Jolla, CA). Where data were known or predicted to violate assumptions for parametric statistical testing (not sampled from populations that follow a Gaussian distribution and/or unequal variances between groups), an equivalent non‐parametric test was performed. Data between 2 groups were analyzed using either the unpaired *t* test or Mann–Whitney *U* test with appropriate correction for multiple comparisons using the Holm–Sidak and Bonferroni methods, respectively. Statistical comparisons for > 2 groups were analyzed using either analysis of variance followed by Holm–Sidak multiple comparison test or Kruskal–Wallis followed by Dunn's multiple comparison test between groups. Histological analyses of spinal cord regions of interest were analyzed separately in light of known pathophysiology of disease. For longitudinal analyses, multiple regression with cluster analysis and robust standard errors (Stata v12; StataCorp, College Station, TX) was used to assess the effect of the test variable while taking into account the effect of other variables including disease model (YG8R), BMT, and age as well as correlation between longitudinal replicates from the same individual. For all tests, values of *p* < 0.05 were considered statistically significant. Statistical tests were all 2‐sided. Data are represented as mean ± standard error of the mean (SEM).

## Results

To investigate the therapeutic effects of BMT, we used 3‐month YG8R transgenic mice. These mice are frataxin‐deficient and develop progressive neurodegeneration and cardiac pathology.^16^, ^21^, ^30^

### Detection of EGFP‐BM Chimerism and Mobilization of PB Mononuclear Cells in Mice with FA

We successfully established chimeric (wild‐type control or YG8R) mice stably reconstituted with EGFP‐expressing BM cells containing wild‐type copies of the *Fxn* gene (see Fig 1). At 6 weeks post‐transplantation, approximately 95% of nucleated cells within the PB of transplanted mice were EGFP‐positive and thus donor‐derived. No significant change in the levels of EGFP‐positive cells in the PB were observed at 20‐weeks posttransplant (data not shown). More than double the number of circulating mononuclear cells (MNCs) were present in PB of transplanted compared to non‐transplanted YG8R mice. By contrast, BMT in wild‐type controls did not result in an increased circulating MNC number when compared to age‐matched non‐transplanted controls.

### BMT of Cells That Stably Express Wild‐Type Fxn Improves Neurobehavioral Deficits

**Figure 2:**
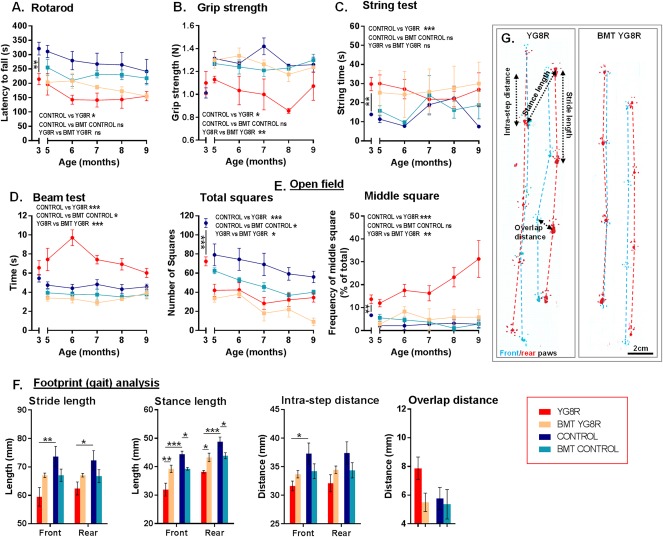
Improvements in neurobehavioral deficits in YG8R mice after allogeneic bone marrow transplantation (BMT). (A–E) Longitudinal results for (A–D) motor performance and locomotor performance (E; open field test) in mice from 3 to 9 months of age. (F) Footprint (gait) analysis and (G) representative footprint traces in mice 9 months of age. For A–E, statistical analysis employed a regression model to determine whether there was an independent significant effect of disease model (YG8R) or BMT on behavioral score. Three‐month comparisons (pre‐transplant; control vs YG8R; A–E) were analyzed using the unpaired *t* test, with the exception of open field (middle square), where the Mann–Whitney *U* test was employed. Statistical comparisons of control versus YG8R, YG8R versus BMT YG8R, and control versus BMT control for footprint analysis (F) were compared using analysis of variance followed by Holm–Sidak multiple comparisons test. *p < 0.05, **p < 0.01, ***p < 0.001, ns = not significant; values represent mean ± standard error of the mean. For all tests, n = 4 (BMT YG8R), all other groups n = 5.

Motor coordination and locomotor activity in both transplanted and non‐transplanted mice was assessed monthly for 6 months. At 3 months of age (before therapeutic intervention) and throughout the duration of the study, neurological deficits were apparent in the non‐transplanted YG8R mice compared to age‐matched non‐transplanted wild‐type controls (Fig 2).

BM‐transplanted YG8R mice showed significant improvements in grip strength tests, beam walk tests, open field tests (middle square), and gait analyses, compared to untreated YG8R mice (see Fig 2). No overall improvements were seen in either the rotarod or string test. Transplant had a negative effect on open field (total squares) performance. BMT in wild‐type control mice resulted in no improvements in performance when compared to age‐matched non‐transplanted wild‐type controls, with the exception of a small but significant improvement in the beam walk test. Conversely, some decline in performance was also evident in the open field (total squares) and gait analysis. As a deterioration in locomotor performance using the open field test (total squares) was observed in both transplanted YG8R and control mice, it is likely this effect was caused by high‐dose total body irradiation–induced tissue injury during myeloablative conditioning.^31^

### BMT Increases Frataxin and Antioxidant Defenses

Using immunoblotting techniques, we measured protein levels of both endogenous human frataxin (expressed by the humanized YG8R mouse) and total frataxin (mouse frataxin [wild‐type transplant‐derived frataxin] + human frataxin) in the cerebellum of YG8R mice (aged 9 months). Frataxin protein levels were calculated by normalizing to levels of internal reference proteins β‐actin and NeuN detected on the same blot. BMT in YG8R mice resulted in a marked increase in total frataxin expression; human frataxin expression was elevated, but not significantly. Notably, in all cases, increases in frataxin expression were more prominent when normalized to the neuronal‐specific marker NeuN than when normalized to ubiquitously expressed β‐actin, suggesting that BMT, at least in part, potentiates increases in neuronal frataxin (Table).

Frataxin deficiency results in dysregulation in cellular antioxidant defenses,^32^ with deficiencies in key antioxidant regulators including PGC‐1alpha and Nrf2.^33^, ^34^ BMT in YG8R mice was associated with significant increases in PGC‐1alpha expression. No significant increases in the expression of the antioxidants SOD1, SOD2, catalase, and glutathione peroxidase‐1 were apparent (see Table).

### BMT Reverses FA‐Associated Pathology

**Figure 3:**
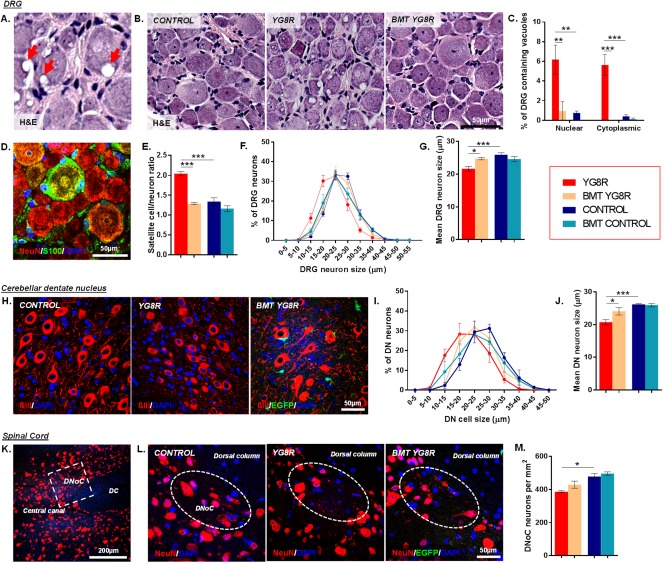
Bone marrow transplantation (BMT) reverses Friedreich's ataxia–associated pathology. (A) High‐powered image of hematoxylin and eosin (H&E)‐stained dorsal root ganglion (DRG) neurons containing vacuoles *(arrows)*. (B) DRG depicting reductions in vacuolization of the large sensory neurons within transplanted YG8R mice. (C) The frequency of DRG neurons containing nuclear or cytoplasmic vacuoles. (D) Typical DRG histology with satellite glial cells (green) covering the surface of DRG sensory neurons (red). (E) The DRG satellite‐to‐neuron cell ratio. (F, G) The size range (F) and mean cell size (diameter; G) of DRG neurons. (H) High‐powered images of ßIII‐tubulin–labeled neurons within the cerebellar dentate nucleus (DN). (I, J) The size range (I) and mean cell size (diameter; J) of cerebellar DN neurons. (K, L) Representative low‐powered (K) and high‐powered (L) images of NeuN‐positive neurons within the dorsal nucleus of Clarke (DNoC). (M) The number of DNoC neurons/mm^2^. All statistical comparisons of control versus YG8R, YG8R versus BMT YG8R, and control versus BMT control mice were analyzed using analysis of variance followed by Holm–Sidak multiple comparisons test. *p < 0.05, **p < 0.01, ***p < 0.001; values represent mean ± standard error of the mean. For all tests, n = 4 (BMT YG8R), all other groups n = 5. DAPI = 4′,6′‐diamidino‐2‐phenylindole; DC = dorsal column; EGFP = enhanced green fluorescent protein.

**Figure 4:**
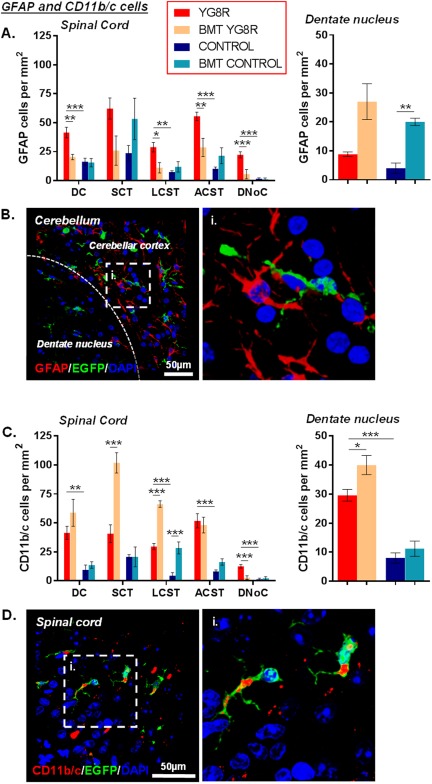
Bone marrow transplantation (BMT) alters glial/immune cell infiltration to areas of tissue injury. (A, C) The number of glial fibrillary acidic protein (GFAP)‐positive (A) and CD11b/c‐positive (C) cells/mm^2^ within the spinal cord or cerebellar dentate nucleus. (B, D) Images of GFAP‐positive (B) and CD11b/c‐positive (D) cells within the cerebellar dentate nucleus or spinal cord. Statistical comparisons of control versus YG8R, YG8R versus BMT YG8R, and control versus BMT control mice were analyzed using analysis of variance followed by Holm–Sidak multiple comparisons test (A [spinal cord], C) or Kruskal–Wallis followed by Dunn's multiple comparison test (A [dentate nucleus]). *p < 0.05, **p < 0.01, ***p < 0.001; values represent means ± standard error of the mean. For all tests, n = 4 (BMT YG8R), all other groups n = 5. ACST = anterior corticospinal tract; DAPI = 4′,6′‐diamidino‐2‐phenylindole; DC = dorsal column; DNoC = dorsal nucleus of Clarke; EGFP = enhanced green fluorescent protein; LCST = lateral corticospinal tract; SCT = spinocerebellar tract.

YG8R mice showed vacuolar degeneration of large sensory DRG neurons, loss of neurons within the dorsal nucleus of Clarke (DNoC), and degeneration of neurons of the cerebellar dentate nucleus, associated with astrogliosis and microglial infiltration (Figs 3 and 4), all changes that closely mimic those of human FA.^3^, ^5^ BMT in YG8R mice completely attenuated intracellular (nuclear and cytoplasmic) vacuolar degeneration of the large sensory neuronal cell bodies, and this change was associated with a reduced satellite cell‐to‐DRG neuron ratio, a likely consequence of improved large sensory neuronal cell survival (see Fig 3).^3^ Within the cerebellar dentate nucleus, mean neuronal size was increased, indicating preservation of large neuronal cells. Alternatively, we found no significant preservation of neurons in spinal cord DNoC of transplanted mice.

BMT in YG8R mice resulted in reduced numbers of GFAP‐positive astrocytes in the spinal cord (see Fig 4). CD11b/c‐positive macrophages/microglial cells in both the spinal cord and cerebellar dentate nucleus were increased in transplanted YG8R mice. BMT in wild‐type controls resulted in elevated GFAP and CD11b/c cell numbers in the cerebellar dentate nucleus and the spinal cord lateral corticospinal tract, respectively, when compared to age‐matched non‐transplanted controls.

### Large Numbers of BM‐Derived Cells Infiltrate the Nervous System of YG8R Mice after BMT

**Figure 5:**
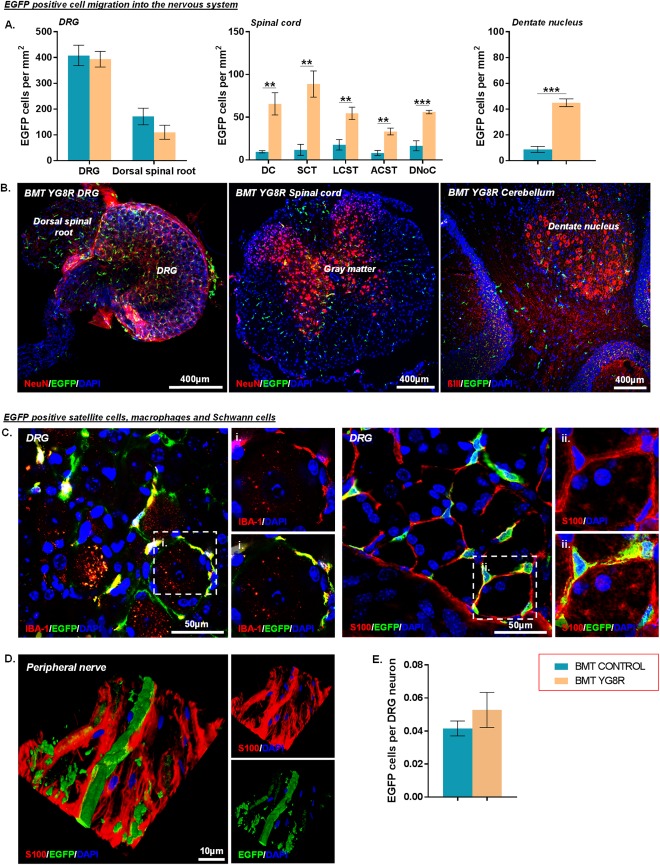
Bone marrow transplantation (BMT) results in large numbers of enhanced green fluorescent protein (EGFP)‐expressing bone marrow–derived cells migrating and integrating into the nervous system. (A, B) The frequency (A) and representative images (B) of EGFP‐positive cells within the nervous system of transplanted mice. (C) EGFP‐expressing cells labeled with either S100 or IBA‐1 within dorsal root ganglia (DRG). The hatched areas depict the locations of the higher‐powered images. (D) An EGFP‐expressing S100‐positive Schwann cell wrapping around an axon. (E) The frequency of EGFP cells wrapping each DRG neuron. All statistical comparisons of BMT control versus BMT YG8R were analyzed using the unpaired *t* test. Correction for multiple comparisons within each anatomical location were made using the Holm–Sidak method. **p < 0.01, ***p < 0.001; values represent mean ± standard error of the mean. For all tests, n = 4 (BMT YG8R), all other groups n = 5. ACST = anterior corticospinal tract; DAPI = 4′,6′‐diamidino‐2‐phenylindole; DC = dorsal column; DNoC = dorsal nucleus of Clarke; LCST = lateral corticospinal tract; SCT = spinocerebellar tract.

We detected significant numbers of EGFP‐positive cells within DRG, peripheral nerves, spinal cord, and cerebellum of YG8R mice transplanted with EGFP‐expressing BM cells (Fig 5). The frequency of EGFP‐expressing cells was significantly higher within the spinal cord and cerebellar dentate nucleus of transplanted YG8R mice compared to transplanted wild‐type controls. CD11b/c–positive or IBA‐1–positive macrophages/microglia were, as expected (being of myeloid origin), found to co‐express EGFP in both wild‐type and YG8R transplanted mice (see Figs 4D and 5C). EGFP‐positive cells did not co‐express the astrocytic marker GFAP in spinal cord or cerebellum (see Fig 4B). However, significant numbers of cells expressed the satellite/Schwann cell marker S100 within the DRG and peripheral nerves, respectively (see Fig 5). The morphological characteristics of these cells matched those of typical S100 cells in situ within their given anatomical locations. The frequency of EGFP‐positive cells surrounding the DRG neurons was the same in transplanted YG8R and age‐matched transplanted control mice.

### Fusion of BM‐Derived Cells within the Nervous System of YG8R Mice

**Figure 6:**
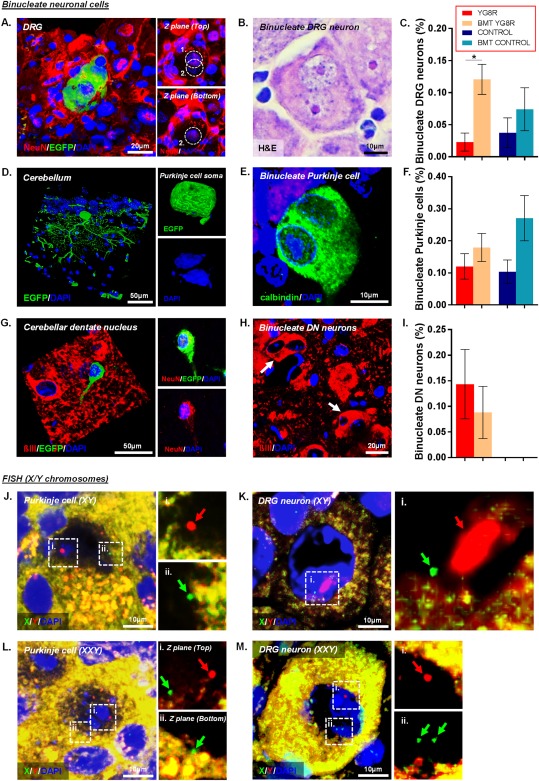
Bone marrow transplantation (BMT) results in enhanced green fluorescent protein (EGFP) bone marrow (BM)‐derived cells fusing with neurons throughout the nervous system of YG8R mice. (A) An EGFP‐expressing binucleate dorsal root ganglion (DRG) neuron labeled with NeuN. The hatched areas in the smaller images depict the locations of the 2 distinct nuclei. (B) A binucleate DRG neuron visualized using hematoxylin and eosin (H&E) staining. (D) An EGFP‐expressing binucleate Purkinje cell. (E) A binucleate Purkinje cell visualized using calbindin‐D28K/4′,6′‐diamidino‐2‐phenylindole (DAPI) labeling. (G) An EGFP‐expressing cell in the cerebellar dentate nucleus (DN). The smaller images depict the same cell coexpressing NeuN. (H) ßIII‐tubulin–expressing binucleate cells *(arrows)* within the cerebellar DN. (C, F, I) The percentage of binucleate DRG neurons (C), Purkinje cells (F), and cerebellar DN neurons (I) in non‐transplanted and transplanted mice. (J, K) Fluorescent in situ hybridization (FISH) analysis in BMT YG8R mice, using fluorescently labeled X (green) and Y (red) chromosomal probes, shows the presence of the male donor Y chromosome in the nucleus of both Purkinje cells (J) and DRG neurons (K). (L, M) Purkinje cell (L) and DRG neuron (M) heterokaryons in BMT YG8R mice containing 3 distinct chromosomes (XXY), providing evidence for fusion between male wild‐type BM‐derived cells and female YG8R host neurons. Hatched areas indicate the locations of the higher‐powered images without DAPI labeling. Statistical comparisons of control versus YG8R, YG8R versus BMT YG8R, and control versus BMT control mice were analyzed using analysis of variance followed by Holm–Sidak multiple comparisons test (C, F) or Kruskal–Wallis followed by Dunn's multiple comparison test (I). *p < 0.05; values represent mean ± standard error of the mean. For all tests, n = 4 (BMT YG8R), all other groups n = 5.

We found EGFP‐expressing BM‐derived cells integrated with DRG neurons to form binucleate NeuN‐positive cells (Fig 6). The frequency of these binucleate DRG neurons was significantly higher in transplanted YG8R mice than in non‐transplanted YG8R mice. We also found EGFP‐positive cells within the cerebellum of the YG8R mouse associated with typical binucleate Purkinje cell heterokaryons. EGFP‐positive cells, with typical neuronal morphology, expressing both the neuronal markers NeuN and ßIII‐tubulin, were identified (albeit very rarely) within the cerebellar dentate nucleus. A proportion of these cells were also binucleate.

### Evidence for Fusion and Genetic Integration between Wild‐Type BM‐Derived Cells and Endogenous Neurons in YG8R Mice

Utilizing sex‐mismatched BMT (transplanting male donor wild‐type BM cells into female YG8R mice; see Fig 1B), the presence of male BM‐derived cells and/or nuclei in the female YG8R brain was detected using in situ hybridization with fluorescently labeled X and Y chromosomal DNA probes (see Fig 6). Cerebellar and DRG sections were selected for examination, and Purkinje cells or DRG neurons, respectively, were analyzed for their sex chromosome content. Using laser scanning confocal microscopy, the Y chromosome was readily detected scattered throughout the tissue. In a number of Purkinje cells and DRG neurons, identified by their unique morphology and location, a Y chromosome was detected within the nucleus (nuclei did not always contain 2 sex chromosomes due to the thickness of the sections [8 µm] encompassing less than half of the entire cell soma). Furthermore, several Purkinje cells and DRG neurons were shown to contain 3 or 4 distinct chromosomes (XXY or XXXY), providing evidence for fusion and donation of nuclear genetic material between male wild‐type BM‐derived cells and female YG8R host neurons.

### G‐CSF and SCF Administration in Conjunction with BMT Further Increases BM Cell Integration into the Cerebellum and DRG of YG8R Mice

**Figure 7:**
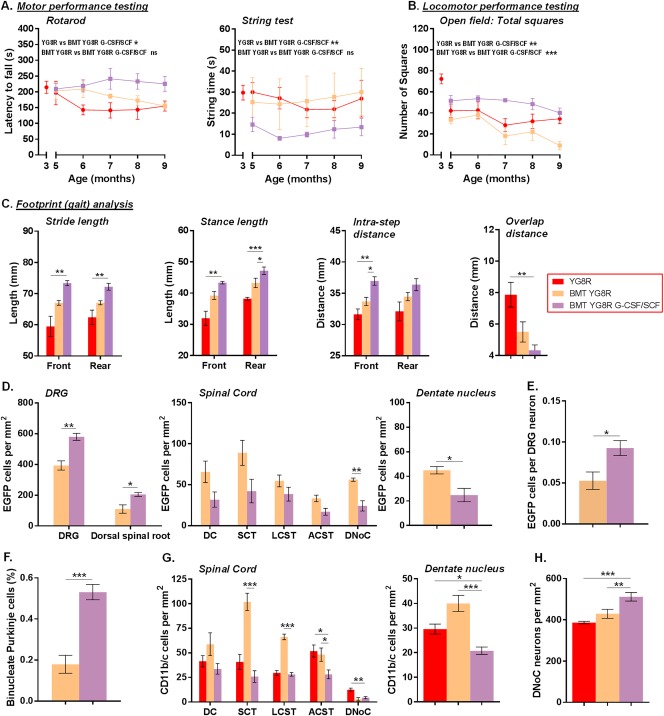
Granulocyte‐colony stimulating factor (G‐CSF) and stem cell factor (SCF) administration improves behavioral and anatomic parameters in YG8R mice that have received a bone marrow transplant (BMT). (A, B) Longitudinal results for (A) motor performance (rotarod and string test; A) and locomotor performance (open field test: total squares; B) in mice from 3 to 9 months of age. (C) Footprint (gait) analysis in mice 9 months of age. (D–G) The number of enhanced green fluorescent protein (EGFP) cells/mm^2^ within the nervous system (D), the frequency of EGFP cells wrapping each dorsal root ganglion (DRG) neuron (E), the percentage of binucleate Purkinje cells (F), and the number of CD11b/c‐positive cells/mm^2^ within the spinal cord and dentate nucleus (G). (H) The number of dorsal nucleus of Clarke (DNoC) neurons/mm^2^. Statistical comparisons of YG8R versus BMT YG8R G‐CSF/SCF and BMT YG8R versus BMT YG8R G‐CSF/SCF were analyzed using multiple linear regression (A, B), unpaired *t* test (correction for multiple comparisons within each anatomical location were made using the Holm–Sidak method; D, E, F), or an analysis of variance followed by Holm–Sidak multiple comparisons test (C, G, H). Multiple regression was employed to determine whether there was an independent significant effect of cytokine administration (in transplanted YG8R mice) or BMT + cytokine administration (in YG8R mice) on behavioral score. *p < 0.05, **p < 0.01, ***p < 0.001; values represent mean ± standard error of the mean. For all tests, n = 4 (BMT YG8R), all other groups n = 5. ACST = anterior corticospinal tract; DC = dorsal column; LCST = lateral corticospinal tract; ns = not significant; SCT = spinocerebellar tract.

A subgroup of transplanted YG8R mice were subcutaneously injected with the cytokines G‐CSF and SCF daily for 5 consecutive days, each month for 5 consecutive months (see Fig 1A). Cytokine administration stimulated mobilization of MNCs approximately 3‐fold in transplanted YG8R mice (3.35 ± 0.29 × 10^7^ cells/ml [BMT YG8R] vs 8.71 ± 0.11 × 10^7^ cells/ml [BMT YG8R G‐CSF/SCF], mean ± SEM, n = 4 and n = 5, respectively, *p* < 0.001, unpaired *t* test). G‐CSF/SCF administration in transplanted mice also led to significant improvements in all motor coordination and locomotor activity tests when compared to non‐transplanted YG8R mice (including the rotarod, string test and open field test [Fig 7A–C], where no initial improvements in performance were evident when YG8R mice received a transplant alone [see Fig 2]). Only in the open field test (total squares) and gait analysis did G‐CSF/SCF administration in transplanted mice lead to a significant improvement in performance over YG8R mice that had received a transplant alone (see Figs 7B and 7C).

G‐CSF/SCF administration increased frequencies of EGFP‐positive cells within DRG and dorsal spinal roots, but decreased frequencies within the spinal cord DNoC and dentate nucleus (see Fig 7). Furthermore, it led to an increased frequency in both EGFP‐expressing cells surrounding DRG neurons and binucleate Purkinje cells (3‐fold) within the cerebellum. No changes in the frequency of GFAP‐expressing cells were observed with the addition of G‐CSF/SCF (data not shown); however, CD11b/c macrophages/microglia numbers were significantly reduced in number within both the spinal cord and dentate nucleus. We also found a significant preservation of neurons in spinal cord DNoC of transplanted mice that had received G‐CSF/SCF. Of note, G‐CSF/SCF administration had no adverse effect on any outcome analyzed within this study.

## Discussion

Here we provide evidence that transplantation of BM cells expressing normal copies of the frataxin gene (*Fxn*) in a humanized mouse model of FA leads to significant functional, biochemical, and pathological improvements.

Tissue repair is thought to involve the selective recruitment of stem and progenitor cells, both local and including those derived from the BM.^35^, ^36^ Post‐BMT, YG8R mice displayed a disease‐related mobilization of endogenous MNCs from the BM niche into the PB. Transplanted control mice showed no signs of elevated circulating MNCs, providing possible insights into how "healthy" BM‐derived cells respond in non‐pathological/pathological conditions. Impaired mobilization of progenitor cells may be inherent to the disease^37^; in support of this, we have previously reported a deficiency in neural precursors found throughout the nervous system of YG8R mice.^16^

EGFP‐expressing BM cells expressed wild‐type frataxin, and neurological improvements may relate to the elevated levels of frataxin within the nervous system. Our protein expression and histological studies support an increase in neuronal frataxin levels post‐transplant. BMT also increased expression of PGC‐1alpha, a key regulator of cellular redox homeostasis associated with frataxin deficiency.^38^ Re‐establishing normal cellular function through increasing frataxin levels holds therapeutic promise. This has been demonstrated experimentally using gene therapy approaches, where increasing cellular frataxin levels through viral delivery of a *FXN* transgene led to robust correction of cellular parameters in models of FA,^39^, ^40^ in both cell culture and myocardial tissue.

BMT in YG8R mice resulted in stable long‐term engraftment of EGFP‐positive BM cells with extensive integration of EGFP‐expressing BM‐derived cells into areas of FA‐associated nervous tissue injury, namely DRG, the spinal cord, and cerebellum.^41^ Studies have previously reported that intrathecally injected in vitro culture‐expanded mesenchymal stem cells (MSCs) can migrate into the DRG of FA mice and exert a neurotrophic effect.^42^ During BMT, small numbers of donor MSCs are co‐infused with injection of BM MNCs; however, they do not replace recipient MSCs despite complete engraftment of the donor wild‐type hematopoietic system.^43^ This lack of MSC engraftment post‐BMT implies that it is unlikely donor‐derived wild‐type MSCs afford the therapeutic effects of BMT in YG8R mice.

In our study, EGFP‐positive cells expressed NeuN and S100 within the dorsal spinal root and/or DRG, indicating that BM‐derived cells contribute genetic material to neuronal, satellite cell, and myelinating Schwann cell populations. Both dysfunctional frataxin‐deficient neurons and glia are likely to contribute to neuropathology in FA.^6^, ^7^ Pathology in FA occurs primarily in the DRG, with significant but independent changes to both the large sensory neurons and surrounding satellite cells, ultimately resulting in neuronal atrophy.^5^ Replacement of these dysfunctional cell types within the DRG of transplanted mice, which under normal conditions provide bidirectional trophic support to one another,^5^ may improve the survival of DRG neurons. EGFP‐positive cells surrounding the DRG neurons also expressed the macrophage marker IBA‐1. Following nerve damage, neuron–macrophage interactions can result in the acquisition of a pro‐regenerative phenotype in macrophages, potentially enhancing the axonal regenerative capacity.^44^ There are also reports that BM‐derived macrophages can become satellite‐like cells following nerve injury, enveloping DRG neurons and modifying the neuronal environment through regulation of neurotrophin synthesis.^45^

BM‐derived cells fusing with neurons following BMT, to form binucleate heterokaryons, has been observed in humans.^9^ Moreover, pathophysiological processes associated with neurological injury in FA appear to augment these fusion events.^46^ Experimentally, in mice that have received BMT, BM‐derived cells fuse with cerebellar Purkinje cells to form stable heterokaryons.^47^ Observations from these studies have suggested that BM cells fusing with neurons and donating their genetic material represent a biological cell‐mediated mechanism for neuronal protection in the adult brain. EGFP‐expressing binucleate heterokaryons within both the cerebellum (cerebellar dentate nucleus neurons and Purkinje cells) and DRG of YG8R mice revealed significant nuclear integration of transplanted cells into nervous system tissue. Moreover, sex‐mismatched BMT (transplanting male donor BM cells into female YG8R mice) and detection of the Y chromosome provided definitive evidence for cell fusion and donation of nuclear material between donor BM‐derived cells and host YG8R neurons. Cellular fusion has previously been noted in cerebellar Purkinje cells^47^ and spinal cord motor neurons.^48^ The novel identification of EGFP‐expressing binucleate DRG and cerebellar dentate nucleus neurons, in addition to sex chromosome analysis, widens the range of cell types in which fusion has been reported. We also show that BMT alone increased the frequency of cell fusion within the DRG. Each fused binucleate cell within transplanted mice, in theory, contains a corrected form of the *Fxn* gene. Genes derived from the wild‐type–donated nucleus are clearly present and expressed within the YG8R host cell, demonstrated through translation of the *EGFP* transgene, providing novel proof of concept of BMT gene therapy within the nervous system. In organs other than the brain, such as the liver, BM cell migration and integration represent an important process by which degenerating genetically damaged cells can be rescued.^14^, ^15^

We recently demonstrated that the BM stem cell–mobilizing cytokines G‐CSF and SCF display strong neuroprotective effects in the YG8R mouse model (without prior BMT).^16^ Here, we show that the combination of G‐CSF and SCF not only displays direct neuroprotective effects in FA,^16^ but also aids the delivery of BM cells to sites of FA‐associated injury, stimulating neural repair. Significantly, we show also that the administration of G‐CSF/SCF to transplanted YG8R mice further mediates improvements in motor coordination, locomotor activity, and FA‐related pathology. In response to G‐CSF/SCF administration, the incidence of both Purkinje cell fusion and EGFP‐expressing DRG satellite‐like cells/macrophages was elevated. Induced migration of BM‐derived cells into the CNS through G‐CSF/SCF exposure is likely due to the biological action of these drugs on the brain microenvironment, or the cells, and not completely dependent on CNS injury or inflammation.^19^, ^20^ Cytokine administration displayed significant anti‐inflammatory properties in both the spinal cord and dentate nucleus of transplanted YG8R mice, which likely also explains reductions in EGFP‐positive cells detected within those anatomical areas after cytokine treatment. Whether BM integration is enhanced through cytokines simply boosting the levels/availability of circulating stem cells, or they have direct effects on migratory cell signaling processes or passage of cells through the blood–brain barrier, is unknown; these hypotheses warrant further investigation.

Currently, there are no effective treatments to slow the progression of FA. Our findings demonstrate the feasibility of allogeneic BMT as a potential "gene replacement" therapy that may reduce or help reverse neurological disability in patients with FA. This procedure has been extensively studied in humans for other diseases, and it must be taken into consideration that, when used clinically, allogeneic transplantation carries significant risks of morbidity and mortality. The use of reduced‐intensity and/or reduced‐toxicity conditioning could be considered to mitigate tissue injury and potential long‐term side effects associated with high‐dose myeloablative conditioning. In conclusion, we have shown that allogeneic BMT offers the prospect of a novel, rapidly translatable, disease‐modifying, and neuroregenerative treatment for FA.

## Author Contributions

K.C.K., M.A.P., N.J.S., and A.W.: conception and design of the study. K.C.K., K.H., J.R., A.J.C., H.R.H., C.M.R., and B.R.B.: acquisition and analysis of data. K.C.K., A.W., and N.J.S.: drafting a significant portion of the manuscript.

## Potential Conflicts of Interest

Nothing to report.

**Table 1:** BMT Elevates Both Frataxin Levels and Antioxidant Defenses in YG8R Mice

Protein	Normalized to	YG8R	BMT YG8R
FA‐associated proteins			
Human frataxin	ß‐actin	1.00 ± 0.120	1.224 ± 0.136
	NeuN	1.00 ± 0.147	1.618 ± 0.279
Total frataxin	ß‐actin	1.00 ± 0.066	1.486 ± 0.182
	NeuN	1.00 ± 0.074	1.882 ± 0.263^a^
Antioxidant regulators			
PGC1alpha	NeuN	1.00 ± 0.157	2.098 ± 0.409^a^
Nrf2	NeuN	1.00 ± 0.151	1.695 ± 0.333
Antioxidant enzymes			
SOD1	NeuN	1.00 ± 0.092	1.956 ± 0.430
SOD2	NeuN	1.00 ± 0.144	1.385 ± 0.227
Catalase	NeuN	1.00 ± 0.082	1.763 ± 0.366
GPX1	NeuN	1.00 ± 0.047	2.144 ± 0.581

The relative protein expression levels of human frataxin, total frataxin, PGC1alpha, Nrf2, and antioxidant enzymes (SOD1, SOD2, catalase, and glutathione peroxidase 1 [GPX1]) within the cerebellum of YG8R mice. Statistical comparisons between YG8R and BMT YG8R were analyzed using (human frataxin, antioxidant regulators) unpaired *t* tests with correction for multiple comparisons using the Holm–Sidak method; or (total frataxin, antioxidant enzymes) Mann–Whitney *U* tests with correction for multiple comparisons using the Bonferroni method. For all tests, n = 4 (BMT YG8R), n = 5 (YG8R).

a *p* < 0.05, values represent means ± standard error of the mean (expressed relative to YG8R).

BMT = bone marrow transplantation; FA = Friedreich's ataxia.
